# Mouse models of mastitis – how physiological are they?

**DOI:** 10.1186/s13006-015-0038-5

**Published:** 2015-04-06

**Authors:** Wendy V Ingman, Danielle J Glynn, Mark R Hutchinson

**Affiliations:** Discipline of Surgery, School of Medicine, The Queen Elizabeth Hospital, University of Adelaide, Woodville, Australia; Robinson Research Institute, University of Adelaide, Adelaide, Australia; Discipline Physiology, School of Medical Sciences, University of Adelaide, Adelaide, Australia

**Keywords:** Mastitis, Mouse models, Inflammation, Infection, Involution, Mammary gland

## Abstract

Lactation mastitis is a common, but poorly understood, inflammatory breast disease that is a significant health burden. A better understanding of the aetiology of mastitis is urgently required, and will assist in the development of improved prevention and treatment strategies in both human and animal species. Studies in mice have the potential to greatly assist in identifying new drug candidates for clinical trials, and in developing a better understanding of the disease. Mouse models of mastitis involve administration of a mastitis-inducing agent to the mammary gland usually during lactation to examine the host immune response, and progression through to resolution of the disease. There are important variations in the protocols of these mouse models that critically affect the conclusions that can be drawn from the research. Some protocols involve weaning of offspring at the time of mastitis induction, and there are variations in the mastitis-inducing agent and its carrier. Induction of mammary gland involution through weaning of offspring limits the capacity to study the disease in the context of a lactating mammary gland. Administration of live bacteria in an aqueous carrier can cause sepsis, restricting the physiological relevance of the model. Mouse model research should employ appropriately designed controls and closely monitor the health of the mice. In this commentary, we discuss the advantages and study design limitations of each mouse model, and highlight the potential for further development of physiologically relevant mouse models of mastitis.

## Background

Mastitis is a common, but poorly understood, inflammatory breast disease in lactating women that causes localised pain, reduced milk synthesis and the rapid onset of systemic symptoms including fever, flu-like aches, chills and fatigue [1,2]. The challenges posed by this disease lead many women to use supplementary formula, or cease breastfeeding altogether [3-5]. Mastitis in HIV-infected women increases the risk of vertical transmission of HIV to the breastfeeding infant [6,7].

The cause of mastitis is believed to be infection of the breast with bacterial pathogens [8,9]. However, recent research suggests this paradigm might be an oversimplification [10,11]. Milk stasis and maternal stress are strong predisposing factors, and alternative microorganisms such as commensal bacteria [12] and fungi [13] have also been implicated, all of which may trigger or amplify breast inflammation leading to mastitis [14,15]. Therefore, the interactions between inflammatory stimuli including pathogenic bacteria [8] and other components of the microbiome, as well as the host immune response [15,16] are all likely to contribute to shaping the severity of mastitis, duration of symptoms and resolution of the disease. Similarly, it is increasingly recognised that susceptibility and severity of mastitis in dairy cattle is dependent on complex interactions between microorganisms and the host immune response [17]. A better understanding of the aetiology of mastitis is urgently required, and will assist in the development of improved prevention and treatment strategies.

Mouse models of mastitis involve administration of a mastitis-inducing agent to the mammary gland, usually whilst the mouse is lactating, and examination of the host immune response, and progression and resolution of the disease [18,19]. Although the small physical size of mice can pose technical difficulties such as tissue manipulation and sampling, mouse models of disease offer the significant advantage of being time and cost effective over larger animal models. The identical genetic profile of inbred mouse strains reduces variability between animals leading to the ability to generate statistically significant results using a low cohort size. Importantly, the excellent availability of genetically modified mice, antibodies to mouse-specific proteins and other experimental approaches such as adoptive cell transfer enables the identification of new cellular and molecular mechanisms, which can spur translational research leading to clinical trials to treat and prevent mastitis in both human and animal species.

There are a number of important variations in the mouse models employed to study mastitis. Some protocols involve weaning of offspring resulting in mammary gland involution, some administer live pathogens and others administer bacterial products that stimulate inflammation in the absence of active infection. The carrier for the mastitis-inducing agent also varies. In this commentary, we discuss the advantages and study design limitations of each mouse model, and highlight the importance of appropriately designed experimental approaches employing physiologically relevant mouse models of mastitis.

### Permanent removal of pups at mastitis induction

Removal of pups from the lactating female at the time of mastitis induction is necessary if the mastitis-inducing agent is prepared in an aqueous solution, as suckling pups are likely to remove it [20], resulting in reduced and variable disease induction. The permanent removal of offspring from a lactating mother is referred to in the literature as “forced weaning”, and is an experimental approach used to study the biological process of involution [21,22]. Forced weaning results in rapid accumulation of milk in the mammary gland, causing death of alveolar epithelial cells and tissue remodelling, ultimately returning the mammary gland architecture to a non-lactating state [23]. Forced weaning elevates inflammatory mediators in the mammary gland including nuclear factor kappa B [24] and downstream signalling factors such as nitric oxide [25], and there is an influx of macrophages [26,27], all of which are critical components of the cell death and tissue remodelling process.

**Figure 1:**
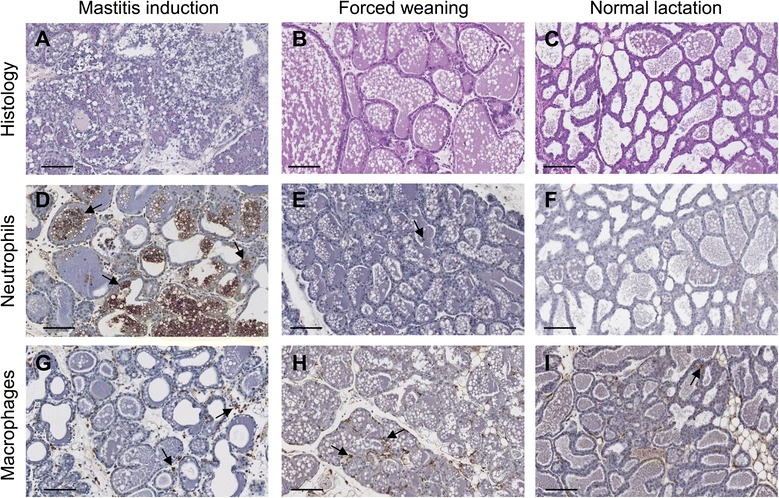
**Alterations in cellular components of the mouse mammary gland during mastitis induction and forced weaning.** Figure shows tissue histology (**A-C**, haematoxylin and eosin stained sections) and abundance of neutrophils **(D-F)** and macrophages **(G-I)** 24 hours after mastitis induction or forced weaning. The macrophages and neutrophils are stained brown, and counter-stained with haematoxylin and indicated by arrows. If the mastitis-inducing agent is administered at the same time as forced weaning, it can be difficult to distinguish the specific inflammatory response to mastitis from the inflammatory response to forced weaning, as both cause alterations in immune cells. Magnification x20, scale bars represent 100 μm. Adapted from Glynn et al. [47] with permission.

Forced weaning of offspring at the time of administration of the mastitis-inducing agent complicates the analysis of mastitis induction, as both involve an influx of immune cells to the mammary gland (Figure 1). Forced weaning also limits the utility of the mouse model, once lactation has ceased there is no longer the capacity to study the progression or resolution of the disease in the context of a functional lactating mammary gland, or the impact of the disease on milk supply. Reduced milk supply is a critical feature of mastitis in women [3,4], and most women continue to breastfeed during episodes of mastitis in line with World Health Organization and Academy of Breastfeeding Medicine recommendations [2,28]. On the other hand, forced weaning results in accumulation of milk within the ducts which is not secreted. This may be a desirable component of the model if the study aims to investigate the contribution of rapid weaning or the oversupply of milk, which are predisposing factors [2], on susceptibility to mastitis.

An alternative approach to forced weaning is to return pups to the lactating dam following mastitis induction. However the optimal time point for this, that does not lead to removal of the mastitis-inducing agent by the suckling pups, has not been established. Some of the complications associated with forced weaning can be overcome through the use of appropriate controls that distinguish inflammation associated with involution from signals associated with mastitis [29]. Despite this, the physiological relevance of such studies becomes limited to the acute phase of inflammation over the first 24 to 48 hours.

### Administration of different mastitis-inducing agents

A variety of mastitis-inducing agents are utilised in mouse models of mastitis, including different strains of live bacteria and fungi, such as *Escherichia coli, Staphylococcus aureus* and *Candida krusei*, and bacterial products such as endotoxin (an outer membrane lipoprotein in Gram-negative bacteria that elicits a strong inflammatory response; also known as lipopolysaccharide) [16,30-32]. Mastitis is inflammation of the breast that may be infective or non-infective [33-35]. Therefore, the particular agent utilised addresses different questions in disease aetiology – the host immune response to an active bacterial infection (live pathogen) compared to the host immune response to an inflammatory stimulus (bacterial product).

Administration of live bacteria to the mammary gland is employed to investigate the immune response to specific infectious bacterial pathogens [30,36] and the efficacy of experimental vaccines [37,38]. These studies often describe the acute response over 24 to 48 hours following mastitis induction and forced weaning [18,19,30,36], although longer term studies in mice with suckling pups have also been described [32,39]. Careful monitoring of mice over the disease time course is essential, as the health of mice administered live bacteria can become severely compromised. For example, an 8°C drop in body temperature occurs within 24 hours [36], and death within 48 hours [40] following administration of some strains of *S. aureus* to the lactating mouse mammary gland, indicative of septic shock. Sepsis is a severe systemic inflammatory response to infection followed by an immunosuppressed state, and is associated with multiple organ failure and loss of haemodynamic control [41]. These systemic events are likely to compromise mammary gland immune system function, altering the expression of pro-inflammatory and anti-inflammatory cytokines, the survival of immune cells and the capacity for antigen presentation. Although mastitis can lead to sepsis in women [42], this is a rare occurrence and does not replicate mastitis disease progression in the majority of clinical cases.

Compared to administration of live bacteria, administration of bacterial products does not have the same capacity to compromise health of mice, however this approach does not enable the study of how the immune system responds to active bacterial infection. Such studies are best utilised to investigate the host inflammatory response, rather than the invading pathogen. The host inflammatory response, including cells, cytokines and intracellular inflammatory mediators, have been implicated in the severity of mastitis in a number of human [43,44], bovine [45,46] and mouse studies [16,47,48], and the therapeutic benefit of dampening this inflammation is currently being explored in mice [49-52]. Therefore, studies that employ administration of bacterial products rather than live bacteria address how the host immune response affects mastitis disease, and can be utilised to explore potential new therapeutics for further development in pre-clinical and clinical studies.

In addition to variations in the mastitis-inducing agent between different studies, the carrier for the agent also varies. The majority of mouse model studies administer the mastitis-inducing agent in an aqueous carrier such as phosphate-buffered saline [18-20,29-32]. Use of an aqueous carrier poses two physiologically relevant problems: (1) the offspring must be removed from the lactating dam to prevent the offspring from suckling and thus removing the mastitis-inducing agent from the mammary gland as discussed above; and (2) the mastitis-inducing agent disperses in the mammary gland causing a widespread mammary gland immune response, that in some instances leads to systemic infection and sepsis. Recently, Matrigel was used as an alternative carrier for deposition of a mastitis-inducing agent into the mammary gland of lactating mice [47]. Matrigel is a gelatinous mixture of proteins that is liquid at 4°C and becomes solid at body temperature. Bacterial endotoxin was combined with Matrigel on ice, and inserted into the teat canal of lactating mice [47], where it formed a solid plug. This feature of Matrigel has a number of physiologically relevant benefits over traditional aqueous carriers, as the mastitis-inducing agent remains localised to a specific region of the mammary gland, thus enabling the continued suckling of pups and analysis of the full course of disease through to resolution, in the context of a lactating mammary gland.

Further development of mouse models utilising Matrigel as a carrier may provide a number of new avenues for exciting research. The cellular and molecular mechanisms that lead to reduced milk supply associated with mastitis can be explored, as pups can continue to suckle throughout the study. Administration of a live pathogen in Matrigel may be less likely to develop into a systemic infection compared to the same pathogen in an aqueous carrier. In addition, many women cease breastfeeding due to mastitis [3-5], and the impact of milk accumulation in the context of acute mastitis could be investigated. However, there are a number of questions regarding the model that are yet to be addressed, including the utility of the model to study mastitis caused by specific strains of live microorganisms. Furthermore, administration of Matrigel in the absence of endotoxin caused an increase in macrophage abundance after 7 days [47]. This is likely to be part of the host response to remove the plug, however it is unknown whether Matrigel clearance affects the host immune response to the stimulus.

## Conclusion

Utilisation of mouse models of mastitis have the potential to greatly assist in the development of new drug treatments for further testing in clinical trials, and to improve our understanding of the relationships between the microbiome, the host immune response and lactation. However, caution should be applied when incorporating knowledge gained in mouse studies to our overall understanding of mastitis. The use of different types of mastitis-inducing agents and carriers affects the conclusions that can be drawn from the research. The physiological limitations of approaches such as forced weaning and systemic infection must also be considered. Appropriately designed research should employ good controls to delineate the immune response to forced weaning from the immune response to mastitis, and closely monitor the health of the mice. Further development of a mouse model that enables continued suckling of pups and stability of mastitis-inducing agents such as live pathogens and other inflammatory stimuli within the mammary gland will assist in the generation of physiologically relevant new knowledge on the development, progression and resolution of mastitis.
